# Integrative identification and preliminary validation of senescence-associated secretory phenotype-related candidate biomarkers in type 2 diabetes

**DOI:** 10.7717/peerj.21729

**Published:** 2026-09-22

**Authors:** Binglian Liu, Jiameng Jia, Nan Wang, Chong Yin, Li Xu, Hongmei Lang

**Affiliations:** 1 Department of General Practice, Sichuan University affiliated Chengdu Second People’s Hospital, Chengdu Second People’s Hospital, West China School of Medicine, Sichuan University, Chengdu, China; 2 School of Clinical Medicine, Qinghai University, Xining, China; 3 Department of Rehabilitation Medicine, Affiliated Hospital of North Sichuan Medical College, Nanchong, China; 4 Department of Ultrasound Medicine, Affiliated Hospital of North Sichuan Medical College, Nanchong, China; 5 Department of Clinical Laboratory, Lab of Nucleic Acid Therapy, Affiliated Hospital of North Sichuan Medical College, Nanchong, China; 6 School of Laboratory Medicine, Translational Medicine Research Center, North Sichuan Medical College, Nanchong, China; 7 Department of Clinical Nutrition, Affiliated Hospital of North Sichuan Medical College, Nanchong, China

**Keywords:** Type 2 diabetes, Senescence-associated secretory phenotype, Biomarkers, Machine learning

## Abstract

Senescence-associated secretory phenotype (SASP)-related inflammatory programs have been implicated in type 2 diabetes mellitus (T2DM), yet blood-based candidates have not been systematically screened and validated across independent cohorts. We adopted standardized senescence signature scoring and multi-cohort independent validation to screen SASP-related candidate genes through literature mining, differential expression analysis, and machine learning, followed by functional enrichment analysis, immune cell composition analysis, and immune cell proportion adjustment. We identified *CCL8* and *MMP9* as key SASP-related candidates; *MMP9* was significantly positively correlated with senescence scores, and both genes were implicated in T2DM progression through E2F targets and interferon gamma response pathways. T2DM led to significant immune microenvironment alterations, and the expression changes of *CCL8* and *MMP9* were independent of peripheral blood mononuclear cell (PBMC) subset frequencies after CIBERSORT adjustment. Expression patterns were consistently validated across all cohorts including the large-scale GSE184050 dataset and single-cell RNA-seq dataset GSE280401, with RT-qPCR providing exploratory support in a small cohort. Collectively, this study provides supportive evidence that CCL8 and MMP9 may represent SASP-related biomarkers in T2DM pathogenesis. Moreover, we present an integrative workflow for systematic screening prioriti zation and validati on of SASP-related candidate genes across multiple cohorts.

## Introduction

Type 2 diabetes mellitus (T2DM) is a chronic metabolic disorder characterized by insulin resistance and progressive pancreatic β-cell dysfunction, manifesting as insufficient insulin secretion and persistent hyperglycemia (Sharjeel et al., 2021). Chronic hyperglycemia induces multisystem complications: approximately 65% of T2DM deaths result from cardiovascular disease, 30%–40% of patients develop diabetic nephropathy, and the risk of diabetic retinopathy is 25-fold higher than in healthy individuals (Sharjeel et al., 2021; Barlow & Mathur, 2022; Shi et al., 2025). Globally, over 530 million T2DM cases were recorded in 2021, with a projected increase to 780 million by 2045. T2DM pathogenesis involves genetic, lifestyle, obesity, and chronic low-grade inflammatory factors (Akash, Rehman & Chen, 2013; Ikegami, Babaya & Noso, 2021; Liu et al., 2006), and is associated with neurodegenerative diseases and infections (DeFronzo, 2004). The “metabolic memory effect” highlights the importance of early intervention (Ceriello, Ihnat & Thorpe, 2009; Yang et al., 2024). Current therapies including metformin, SGLT2 inhibitors, and GLP-1 receptor agonists cannot reverse β-cell dysfunction and carry adverse reactions (Wilcox et al., 2020; Arnold & Buelt, 2022). Identifying key pathogenic genes of T2DM is critical to clarify disease mechanisms and develop targeted therapies.

Cellular senescence, defined as irreversible proliferation arrest induced by stress, is a core hallmark of aging and age-related diseases (Huang et al., 2022; Saul et al., 2022). It is marked by morphological and metabolic alterations, as well as activation of p16INK4a/p21 pathways (Wagner & Wagner, 2022). Senescence detection has evolved from SA-β-gal staining and telomere assays (Itahana, Campisi & Dimri, 2007) to high-throughput omics (Chondronasiou et al., 2022). Landmark studies have established robust senescence research systems: Schafer et al. (2020) developed *in vivo* senescence detection, while Basisty et al. (2020) constructed a comprehensive SASP proteomic atlas, laying methodological foundations for the field.

The senescence-associated secretory phenotype (SASP) comprises bioactive molecules specifically secreted by senescent cells, including cytokines, chemokines, and proteases, which regulate tissue repair, immunity and tumor suppression (Smith et al., 2021; Vinci et al., 2024). SASP genes overlap with immune inflammatory genes, and strict senescence linkage evidence is required to define *bona fide* SASP markers. SASP dysregulation contributes to osteoarthritis, neurodegenerative diseases and other age-related disorders (Coryell, Diekman & Loeser, 2021; Jeon et al., 2018). In T2DM, SASP drives disease progression by triggering chronic inflammation and impairing insulin signaling (Prattichizzo et al., 2016; Midha et al., 2021; Khalid et al., 2021). Leading senescence studies have adopted unbiased proteomics, large cohorts and multi-cohort validation, and Kohli et al. (2021) established a gold-standard senescence scoring algorithm (Schafer et al., 2020; Basisty et al., 2020; Tanaka et al., 2020). Consequently, a comprehensive investigation into the operational mechanisms of SASP in T2DM is highly significant for the advancement of both early diagnostic indicators and treatment approaches for T2DM. However, critical knowledge gaps remain: systematic screening and validation of SASP genes in T2DM are lacking, most studies fail to distinguish SASP from generic inflammation, and few peripheral blood SASP biomarkers are available for clinical T2DM detection.

Using T2DM transcriptomic datasets, this study applied an integrative bioinformatics workflow including differential expression, machine learning, enrichment and immune infiltration analysis to screen SASP-related biomarkers for T2DM. As a confirmatory study, we aimed to validate reported markers within a unified SASP framework and clarify their pathways and immune microenvironment features, with preliminary verification by RT-qPCR. This work provides integrative evidence to improve the diagnosis and therapy of T2DM.

## Materials and Methods

### Data source

Portions of this text were previously published as part of a preprint (Liu et al., 2025). Transcriptome datasets associated with T2DM were retrieved from the Gene Expression Omnibus (GEO) database (https://www.ncbi.nlm.nih.gov/geo/). Specifically, the GSE15932 dataset (platform: GPL570, released in 2012, a microarray dataset) was utilized as the training set for exploratory analyses, including differential expression analysis and machine learning model construction. After excluding samples from patients with other comorbidities, only transcriptomic data from eight blood samples of T2DM patients and eight control samples (healthy subjects) were included. Partial samples in GSE15932 provided available clinical information including age, gender, fasting blood glucose (FBG) and duration of diabetes, while information on medication usage and HbA1c levels was not publicly available. Meanwhile, the GSE21321 (platform: GPL6883) and GSE253502 (platform: GPL16791) datasets were retrieved as independent external validation sets. Among them, the GSE21321 dataset (platform: GPL6883) contained transcriptomic data from nine peripheral blood samples of T2DM patients and eight peripheral blood samples of healthy subjects, with no relevant clinical information available publicly. Notably, GSE21321 (GPL6883) is a peripheral blood expression dataset including adult male participants classified as type 2 diabetes, impaired fasting glucose, and healthy controls based on fasting glucose levels. To compensate for the limitations of single microarray data, a multi-dataset cross-validation strategy (GSE15932 as training set + GSE21321 and GSE253502 as external validation sets) was adopted in this study. Moreover, 83 SASP-related genes (SASP-RGs, derived from senescent cells) were extracted from relevant literature (Table S1) (Saul et al., 2022). A panel of 24 high-confidence senescence biomarkers from Kohli was used to quantify senescent cell burden in all datasets (Kohli et al., 2021). Furthermore, the single-cell RNA-seq dataset GSE280401 (platform: GPL16791) was obtained from the GEO database as an enhanced validation set, including two T2DM PBMC samples and two healthy control samples, which was used to verify the cell-specific expression patterns of candidate biomarkers at single-cell resolution. To strengthen independent validation and address translational relevance, the large-scale cohort GSE184050 (platform: GPL96, 50 T2DM whole blood samples *vs.* 66 healthy controls) was obtained from the GEO database as an additional independent validation set for DEG expression verification.

### Identification of differentially expressed genes

The GSE15932 dataset underwent analysis using the limma package (v3.56.2) to identify differentially expressed genes (DEGs) in comparison between samples from individuals with T2DM and control subjects. In particular, the identification of differentially expressed genes (DEGs) was conducted using a criterion of an absolute log2 fold change (FC) exceeding 0.5, coupled with a statistical significance threshold of *P* < 0.05. The ten most significantly up-regulated and down-regulated genes, as determined by their absolute log2 FC values, were highlighted in the volcano plot and represented in the heatmap visualization.

### Functional analysis of candidate genes

The overlapping genes of SASP-RGs and DEGs were then defined as candidate genes and plotted as venn diagrams with VennDiagram package (v1.7.3). The ClusterProfiler package (v4.15.0.3) was employed to perform Gene Ontology (GO) and Kyoto Encyclopedia of Genes and Genomes (KEGG) analyses. These analyses helped to clarify the biological processes and functions of the candidate genes. Only results with an adjusted *P* <0.05 were considered statistically significant, ensuring the reliability of the identified gene functions and pathways. Additionally, to further investigate the protein-level interactions of the candidate genes, Search Tool for the Retrieval of Interacting Genes/Proteins database (https://string-db.org/) was employed to establish a protein-protein interaction (PPI) network (interaction score > 0.15). High-quality interactions were illustrated through Cytoscape software (v3.1.0).

### Machine learning and validation of biomarkers

To improve the accuracy of identifying SASP-related genes in T2DM, candidate genes were evaluated across the GSE15932 and GSE21321 datasets using machine learning approaches combined with expression level analysis. The SVM-RFE analysis was performed based on the radial basis function kernel (svmRadial). The optimal parameters were determined *via* a strategy combining 5-fold cross-validation and iterative feature subset selection, with the following specific settings: method = “svmRadial”, functions = caretFuncs, method = “cv”, number = 5, and sizes = c(1:num). Parameter optimization adopted the default logic of the caret package (v6.0.94). Moreover, random forest (RF) analysis was centered on the convergence analysis of out-of-bag (OOB) error. A sequence of decision tree numbers (ntree_range = seq(10, 100, by = 2)) was constructed, covering the commonly used range for gene data with small to moderate sample sizes. For each ntree value, a random forest model was built to calculate the corresponding OOB error. The minimum number of features for node splitting (mtry) was determined by combining empirical values with model stability verification: by comparing OOB error rates at different mtry values (3, 5, 7), mtry = 5 was identified as the optimal parameter, as it yielded the lowest OOB error rate and the best accuracy, sensitivity, and specificity in cross-validation. Key parameters for the randomForest::randomForest() function were set as follows: ntree_range <- seq(10, 100, by = 2) (46 candidate values for the number of decision trees, from 10 to 100 with a step size of 2); mtry = 5 (number of randomly selected features for node splitting, fixed temporarily for ntree optimization); importance = TRUE (to calculate feature importance). All machine learning algorithms were evaluated based on accuracy, precision, recall, specificity, and F1-score. We focused on the shared results of the two machine learning algorithms. Based on this, genes that showed significant differences between control and T2DM samples in both GSE15932 and GSE21321 datasets with consistent expression trends were designated as biomarkers. Additionally, an external validation dataset (GSE253502) was further incorporated to verify the stability of their expression.

### Performance prediction of biomarkers

The expression data of biomarkers were extracted based on the GSE15932 dataset, which includes samples from T2DM and control groups. A Least Absolute Shrinkage and Selection Operator (LASSO) regression classification model was constructed *via* glmnet package (v4.3.2), where different *α* values and decision thresholds were iterated, and five-fold cross-validation was adopted. The pROC package (v1.18.0) was used to compute the Receiver Operating Characteristic (ROC) curve, which served as a key metric for evaluating the predictive performance of the model. In addition to assessing the model’s discriminatory ability through the ROC curve, we also evaluated its clinical utility, providing a comprehensive assessment of its practical value in real-world applications. Meanwhile, the data were predicted according to the optimal model parameters, a confusion matrix was generated, and indicators such as accuracy, sensitivity, and specificity were calculated and presented in a visualized graph.

### Gene set enrichment analysis

Within GSE15932 dataset, for exploring signaling pathways associated with biomarkers, GSEA was conducted. Specifically, we conducted Spearman correlation analysis on all samples *via* psych package (v2.1.6), and then genes were sorted by correlation coefficient (cor) in descending order. Then, clusterProfiler package (v4.7.1.3) was utilized to conduct GSEA on each biomarker (|normalized enrichment score (NES)| > 1, false discovery rate (FDR) < 0.25, and *P* < 0.05). Reference gene set “c2.cp.kegg.v2023.1.Hs.symbols.gmt” was obtained from Molecular Signatures Database (MSigDB) (https://www.gsea-msigdb.org/gsea/msigdb). Notably, enrichplot package (v1.18.3) was applied to illustrate the top 5 significant pathways enriched by each biomarker.

### Molecular regulatory networks

To investigate the molecular regulatory mechanisms of biomarkers, microRNAs (miRNAs) targeting them were initially predicted using multiMiR package (v 1.29.0). To ensure the reliability of the prediction, miRNAs with an occurrence frequency of ≥ 4 times were screened from the database. Additionally, transcription factors (TFs) that regulate biomarkers were predicted *via* htfTARGET database (https://www.networkanalyst.ca/). Only those TFs with an occurrence frequency greater than 2 were considered. The networks described above were visualized using Cytoscape (v 3.1.0).

### Analysis of circulating immune cell abundance

To quantify the relative proportions of 22 immune cell subsets, the CIBERSORT algorithm (v0.1.0) was applied to the GSE15932 dataset (Newman et al., 2015). Wilcoxon rank-sum test was performed to compare immune cell proportion differences between T2DM and control groups. Additionally, the ssGSEA algorithm (GSVA v1.42.0) was used to evaluate the abundance of 28 immune cell subsets in GSE15932 and GSE253502 datasets for supplementary verification. Spearman correlation analyses were performed to assess the relationships among immune cells and between biomarkers and immune cells, with |cor| > 0.3 and *P* < 0.05 set as the significance thresholds.

### Single-cell RNA-seq analysis from GSE280401 dataset

The single-cell RNA-seq data of GSE280401 was processed using the Seurat R package (v 5.1.0) (Stuart et al., 2019). First, the expression matrix was generated using the Read10X function, and the Seurat object was constructed with CreateSeuratObject. Quality control (QC) was performed to retain valid cells: (1) genes expressed in >3 cells; (2) cells with 200–4,000 detected genes; (3) cells with <15% mitochondrial gene content; (4) cells with total UMI counts <10,000. After QC, 5278 high-quality cells were retained (filtering rate: 32.3%), with 25,509 genes included.

Data normalization was conducted using the NormalizeData function with the LogNormalize method. Variable genes were identified, and principal component analysis (PCA) was performed using the JackStraw and ElbowPlot functions to determine significant principal components (top 14 PCs, *P* < 0.05). Batch effect correction was performed using Harmony, followed by cell clustering *via* FindNeighbors and FindClusters (resolution = 0.5). Cell type annotation was performed by matching cluster-specific marker genes with the CellMarker 2.0 database and verified by SingleR (Korsunsky et al., 2019; Hu et al., 2023; Aran et al., 2019).

The UMAP algorithm was used to visualize cell clusters and the expression distribution of *MMP9*. Due to *CCL8* being filtered out during QC, only the cell-specific expression of *MMP9* in T2DM and control groups was analyzed.

### Senescence signature scoring analysis

To strictly follow the independent validation paradigm of top senescence studies, GSE15932 was used as the training set, and GSE21321, GSE253502, and single-cell RNA-seq dataset GSE280401 were used as three layers of independent external validation sets. Senescence signature scoring was performed following the standardized method reported by Kohli (Kohli et al., 2021). A panel of 24 high-quality senescence biomarkers was used for scoring. First, the Wilcoxon rank-sum test was used to screen senescence genes with significant differences between groups (*P* < 0.05). The ssGSEA algorithm in the GSVA package (v1.46.0) was applied to calculate the senescence score of each sample. The Wilcoxon rank-sum test was used to compare the differences in senescence scores between the T2DM and control groups. Spearman correlation analysis was performed using the corr.test() function in the psych package (v2.2.9) to explore the relationships between *CCL8*/*MMP9* expression and senescence scores, and the ggcorrplot package (v0.1.4) was used for visualization.

### Assessment of senescent cell burden and correlation with SASP biomarkers

Senescent cell burden was quantified using the standardized senescence score algorithm from Kohli et al. (2021). Briefly, 24 high-quality senescence biomarkers were used, and differentially expressed senescence genes between groups were screened by Wilcoxon rank-sum test (*P* < 0.05). The ssGSEA algorithm in the GSVA package (v1.46.0) was applied to calculate the senescence score of each sample. Spearman correlation analysis was performed using the corr.test() function in the psych package (v2.2.9) to evaluate the relationships between *CCL8*/*MMP9* expression and classic senescence marker genes (p16, p21, p53, LMNB1, DYNLT3). The ggcorrplot package (v0.1.4) was used to visualize the correlation heatmap.

### Immune cell proportion estimation and expression adjustment

Bulk transcriptomic data deconvolution was performed using the CIBERSORT algorithm (v 0.1.0) to estimate the relative proportions of 22 immune cell subsets in the GSE15932 dataset (Newman et al., 2015). Wilcoxon rank-sum test was used to identify significantly different cell subsets between groups (*P* <0.05). To eliminate interference from immune cell proportion changes, ANCOVA was used to re-analyze *CCL8*/*MMP9* expression with cell proportions as covariates. Partial correlation analysis was applied to verify the robustness of correlations between *CCL8*/*MMP9* and senescence score/T2DM status after controlling for immune cell proportions.

### RT-qPCR

Blood of 10 patients with T2DM and 10 healthy individuals was taken at Chengdu Second People’s Hospital. This small sample size was intended for an initial exploratory verification of the bioinformatics findings. The participants successfully filled out and signed an informed consent document, and the ethical approval was obtained from the Ethics Review Committee of Chengdu Second People’s Hospital (Approval No. [KY]PJ2025340). Total RNA was isolated from patient’s blood (1.5 ml each sample) using E.Z.N.A.^®^ Blood RNA Kit (omega Bio-TEK, R6814-02, Winooski, VT, USA) following the manufacturer’s guidelines. RNA concentration and purity were measured using a NanoDrop spectrophotometer, with A260/A280 ratios ranging from 1.8 to 2.0. 1 µg of total RNA was then reverse transcribed using the HiScript^®^ II 1st Strand cDNA Synthesis Kit (Vazyme, R211-02, Nanjing, China) in accordance with the provided protocol (under the conditions of 25 °C for 5 min, 50 °C for 15 min, and 85 °C for 5 min). Quantitative reverse transcription polymerase chain reaction (qPCR) was performed utilizing the ChamQ Universal SYBR qPCR Master Mix (Vazyme, Q711-02-AA, Nanjing, China) alongside a Roche 480 II system (LightCycler 480 II System; Roche, Basel, Switzerland); No template controls (NTC) were included to detect contamination.The total reaction volume was 20 μL. Melting curve analysis was performed to verify the specificity of the PCR products. The cycling conditions were as follows: initial denaturation at 95 °C for 30 s, followed by 40 cycles of 95 °C for 10 s and 60 °C for 30 s. The mRNA expression levels were calibrated against Gapdh mRNA levels *via* the 2-^ΔΔ^CT methodology. The primers were synthesized by Sangon Int (Shanghai, China). The following primers were as follows: *CCL8*, forward 5′-CTTCAAGACCAAACGG-3′ and reverse 5′-GAATCCCTGACCCAT-3′; *MMP9*, forward 5′-GCTACCACCTCGAACTTTGACAG-3′, and reverse 5′-CCCTCAGTGAAGCGGTACATAG-3′; *GAPDH*, forward 5′-ACAACTTTG GTATCGTGGAAGG-3′, reverse 5′-GCCATCACGCCACAGTTTC-3′.

### Statistical analysis

We carried out bioinformatics analyses using the R programming language (v4.3.3). Data across groups were compared by applying the Wilcoxon test. Differences between two groups were analyzed using unpaired t-tests, and results with *P* < 0.05 were regarded as statistically significant. *P* value adjustment was performed for multiple comparisons as needed using the Benjamini–Hochberg (BH) method (for FDR control). Statistical analysis was conducted using GraphPad Prism 9.5.

## Results

### Related functional pathways and PPI network of 7 candidate genes

**Figure 1:**
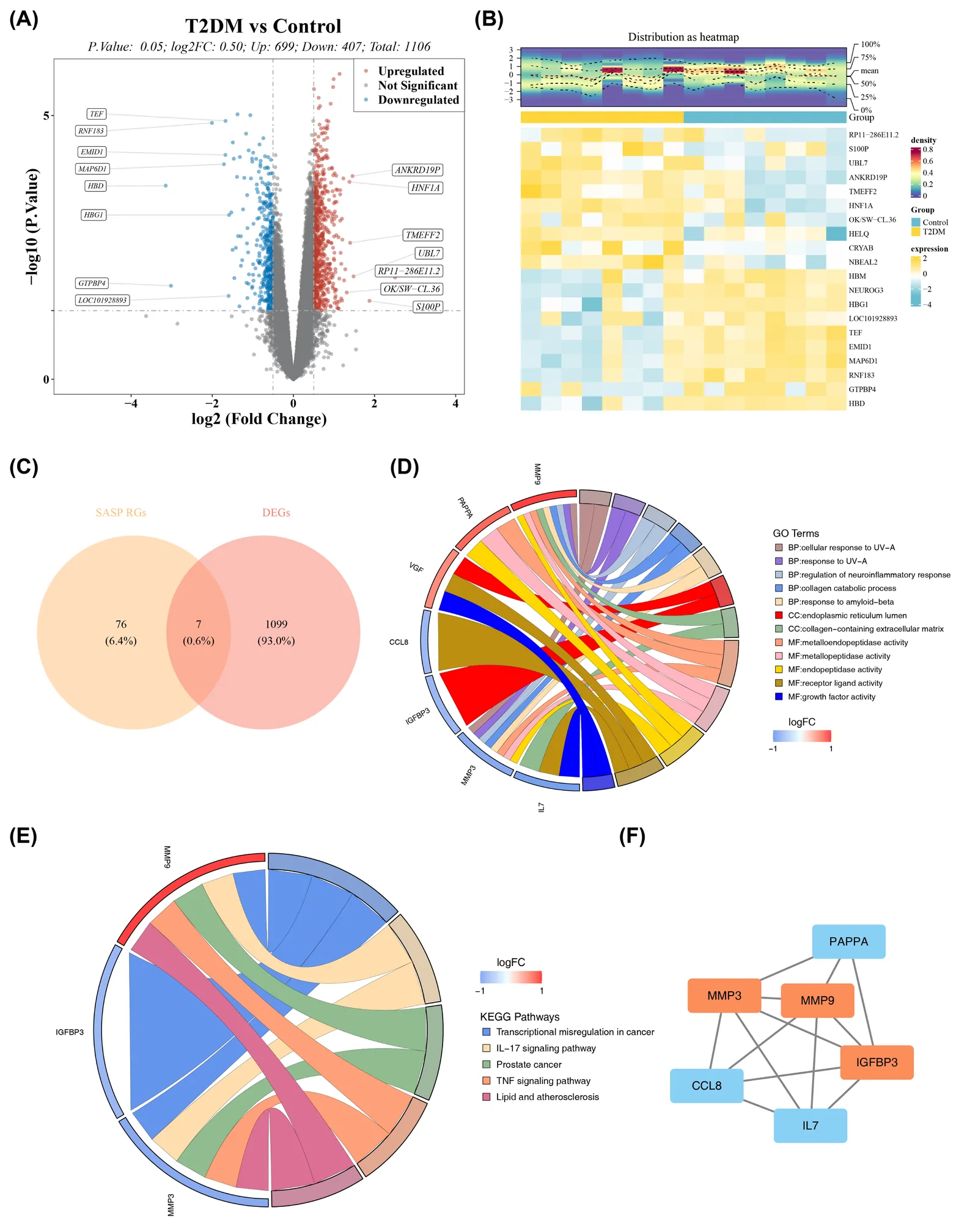
Screening, enrichment analysis, and PPI network of candidate genes. (A–B) The volcano plot and heatmap show 1,106 DEGs between T2DM samples and control samples from the GSE15932 dataset. (C) By intersecting the aforementioned DEGs with 83 SASP-RGs, seven candidate genes were obtained. (D) GO enrichment analysis of the candidate genes. BP: biological processes; CC: cellular components; MF: molecular functions. (E) KEGG pathway enrichment analysis of the candidate genes. (F) PPI network of the candidate genes.

By performing differential expression analysis between T2DM and controls in GSE15932, we identified a total of 1,106 DEGs, along with 699 downregulated and 407 upregulated genes in T2DM (Figs. 1A–1B, Table S2). By crossing DEGs with SASP-RGs, seven candidate genes were obtained (Fig. 1C). Further GO analysis highlighted that the candidate genes were notably enriched across 40 categories, including 23 cellular components (CCs), two molecular functions (MFs), and 15 biological processes (BPs) (Table S3). These terms involved “response to UV-A”, “collagen-containing extracellular matrix”, and “growth factor activity” (adjusted *P* < 0.05), indicating that SASP-RGs might affect the occurrence and development of T2DM at the levels of inflammation, cellular metabolism, signal transduction, and so on (Fig. 1D). Notably, the candidate genes identified, particularly *MMP9* and *CCL8*, are well-documented core effectors of the SASP. *MMP9* is a key protease involved in extracellular matrix remodeling during senescence, while *CCL8* is a chemokine critical for the recruitment of immune cells to clear senescent cells. Furthermore, these factors were linked to various KEGG pathways, including “prostate cancer”, “transcriptional misregulation”, “IL-17 signaling pathway”, “TNF signaling pathway”, and “lipid and atherosclerosis” (adjusted *P* < 0.05; Fig. 1E). These suggested that individuals with T2DM might have a higher risk of developing cancer, or these biomarkers might play an important role in the mutual influence between different diseases. Moreover, the PPI network demonstrated that there were 12 kinds of interactions among the seven candidate genes. Among them, *MMP3*, *MMP9* and *IGFBP3* were connected to the other five proteins (Fig. 1F).

### *MMP9* and *CCL8* were recognized as biomarkers of T2DM

**Figure 2:**
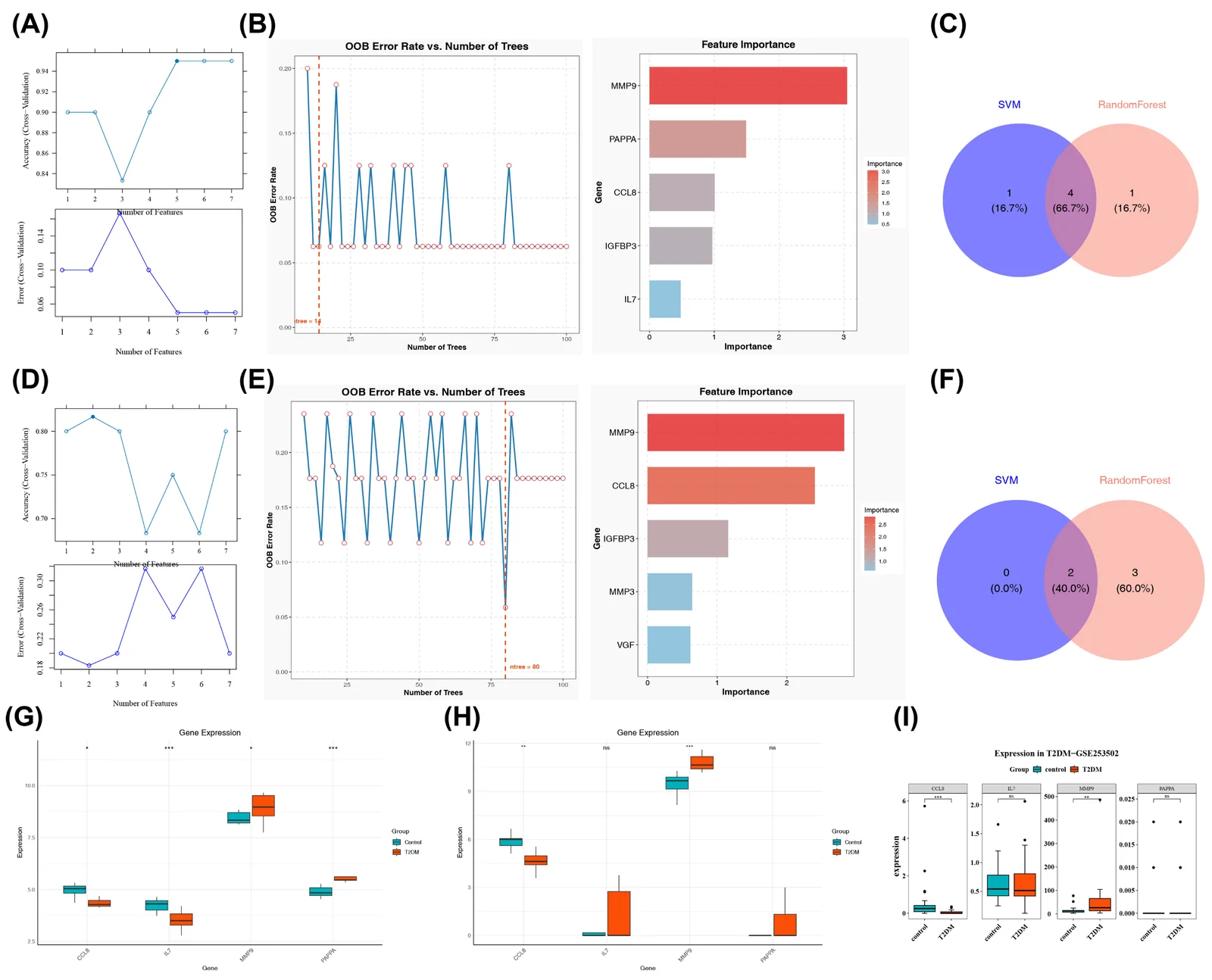
Identification of biomarkers. (A) Cross-validation accuracy and error rate curves of SVM-RFE. The *x*-axis (Number of Features) represents the number of features used in the SVM-RFE feature selection process. (B) Left panel: Relationship between out-of-bag (OOB) error rate and the number of trees in the RF model. The colored dashed line corresponds to “ntree = 14”, indicating a reference point when the number of trees is 14. Right panel: Feature importance of genes, where the *x*-axis (Importance) denotes the feature importance score. (C) Number of contributing genes identified by SVM-RFE and RF analyzes in the training set GSE15932. (D) SVM-RFE analysis in the validation set.(E) RF analysis in the validation set. (F) Number of contributing genes identified by SVM-RFE and RF analyzes in the validation set GSE21321. (G–I) Expression analysis of biomarkers in samples from the training set GSE15932 (G) and validation set GSE21321 (H) and GSE253502 (I). n s, not significant, *p < 0.05, **p < 0.01, ***p < 0.001.

In the training set GSE15932, regarding the seven candidate genes evaluated using SVM-RFE, the results showed that when the SVM-RFE model achieved the lowest error rate, the corresponding five feature genes were *PAPPA*, *IL7*, *MMP9*, *CCL8*, and *MMP3* (Fig. 2A). When mtry = 5, the RF model achieved the optimal performance. A total of 5 RF-derived feature genes were selected: *IL7*, *IGFBP3*, *CCL8*, *PAPPA*, and *MMP9* (Fig. 2B). After intersecting the two sets of feature genes, four common genes were identified (Fig. 2C). Similarly, in the validation set GSE21321, model performance under different feature subsets was evaluated *via* SVM-RFE analysis, and the optimal feature combination consisted of two genes (*MMP9* and *CCL8*) (Fig. 2D). Additionally, both the SVM-RFE and RF models achieved an accuracy, precision, recall, specificity, and F1-score of 1.0. Five genes were identified using the RF model, with *MMP9* and *CCL8* shared between the two machine learning algorithms (Figs. 2E–2F). Subsequently, the Wilcoxon rank-sum test was performed on the GSE15932 and GSE21321 datasets. The results showed consistent expression trends of *MMP9* and *CCL8* across the three datasets. In the T2DM cohort, *MMP9* expression levels were significantly increased, while *CCL8* expression levels were significantly decreased (*P* < 0.05) (Figs. 2G–2H). Furthermore, we further incorporated the GSE253502 dataset with a larger sample size, and the results showed consistent trends (*P* < 0.01), which confirms the stability and reliability of the biomarker expression patterns (Fig. 2I).

### Biomarkers had good predictive performance and were involved in the occurrence of T2DM through multiple pathways

**Figure 3:**
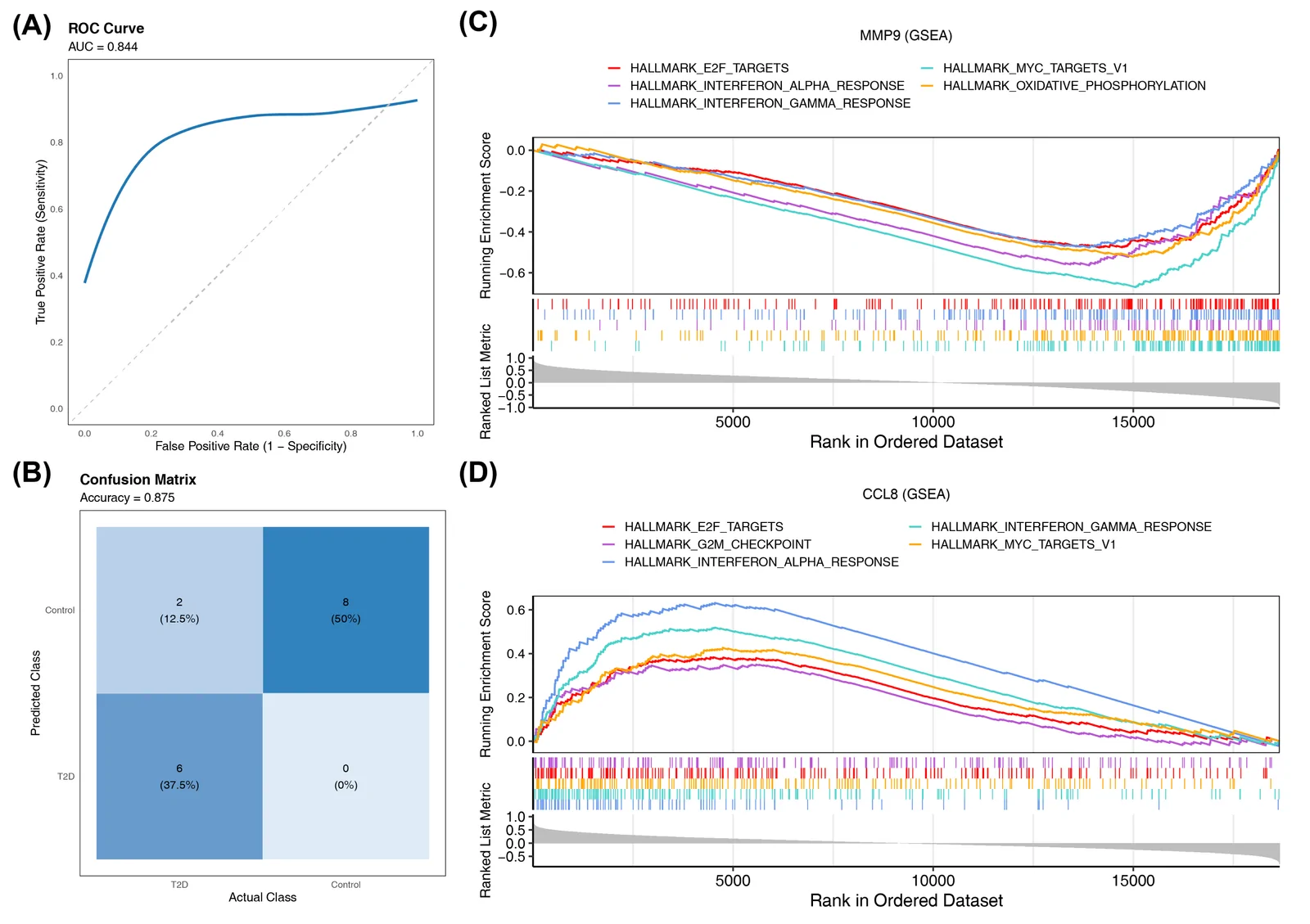
Model prediction and pathway analysis of biomarkers. (A–B) ROC curve and confusion matrix of the model. The closer the AUC value is to 1, the stronger the discriminative ability of the model. In the confusion matrix, the darker the color of a cell, the greater the number of samples it represents; the numerical label in each cell displays the frequency and percentage. (C–D) GSEA enrichment plot displaying the distribution and significance of key pathways.

The optimal parameter combination was determined as alpha = 0.8, lambda = 0.1, and a decision threshold of 0.5, and a model for T2DM classification was trained using these parameters. ROC analysis and the confusion matrix showed that the model had a high discriminative efficiency (AUC = 0.844) and clinical practicality (the identification success rate was 85.7%) (Figs. 3A–3B). Through GSEA, it was found that *MMP9* and *CCL8* were enriched in 23 and 12 pathways, respectively (Table S4). Significantly, the biomarkers exhibited co-enrichment in two essential pathways, namely “E2F targets” and “interferon gamma response” (*P* < 0.05) (Figs. 3C–3D). These pathways indicate that the biomarkers may influence the onset and progression of T2DM *via* the intersection of the “immunity-cell cycle” pathway.

### A variety of molecules could target biomarkers in T2DM

**Figure 4:**
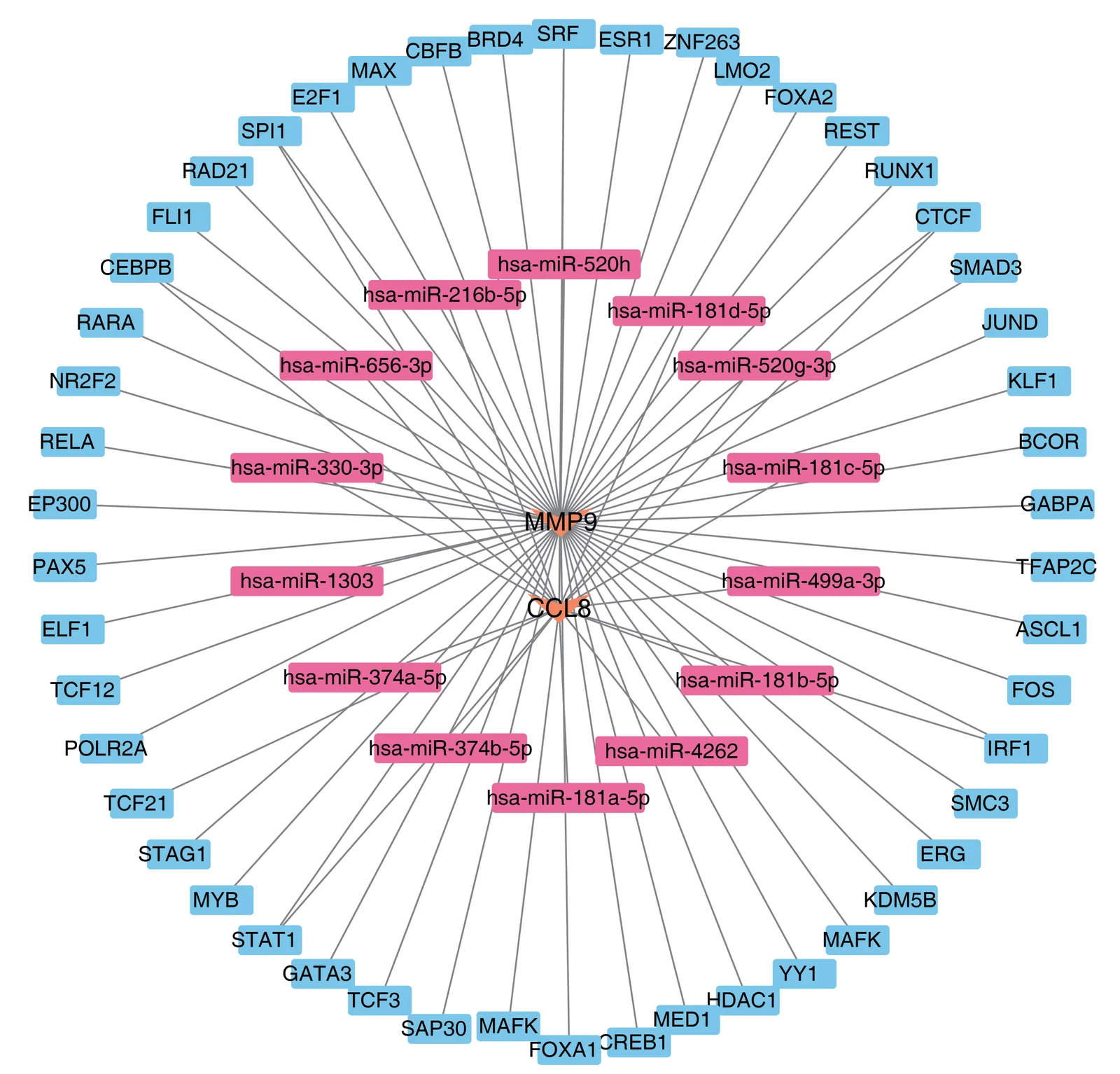
TF-mRNA-miRNA network. Orange diamonds represent the finally identified biomarkers, blue rectangles represent TFs, and red rectangles represent miRNAs. Lines indicate the regulatory relationships between different genes.

The TF-mRNA-miRNA molecular regulatory network showed that SASP-related biomarkers might be involved in a complex regulatory mechanism in T2DM. Through the multiMiR package, two miRNAs (hsa-miR-1303 and hsa-miR-330-3p) that targeted *MMP9* and 13 miRNAs (like hsa-miR-520g-3p and hsa-miR-181a-5p) that targeted *CCL8* were predicted. In addition, 47 TFs, such as CTCF and SPI1, were found to be able to target *MMP9*, and eight TFs, such as MAFK and TCF21, were able to target and regulate *CCL8* (Fig. 4 and Table S5).

### Altered peripheral immune profiles in T2DM

**Figure 5:**
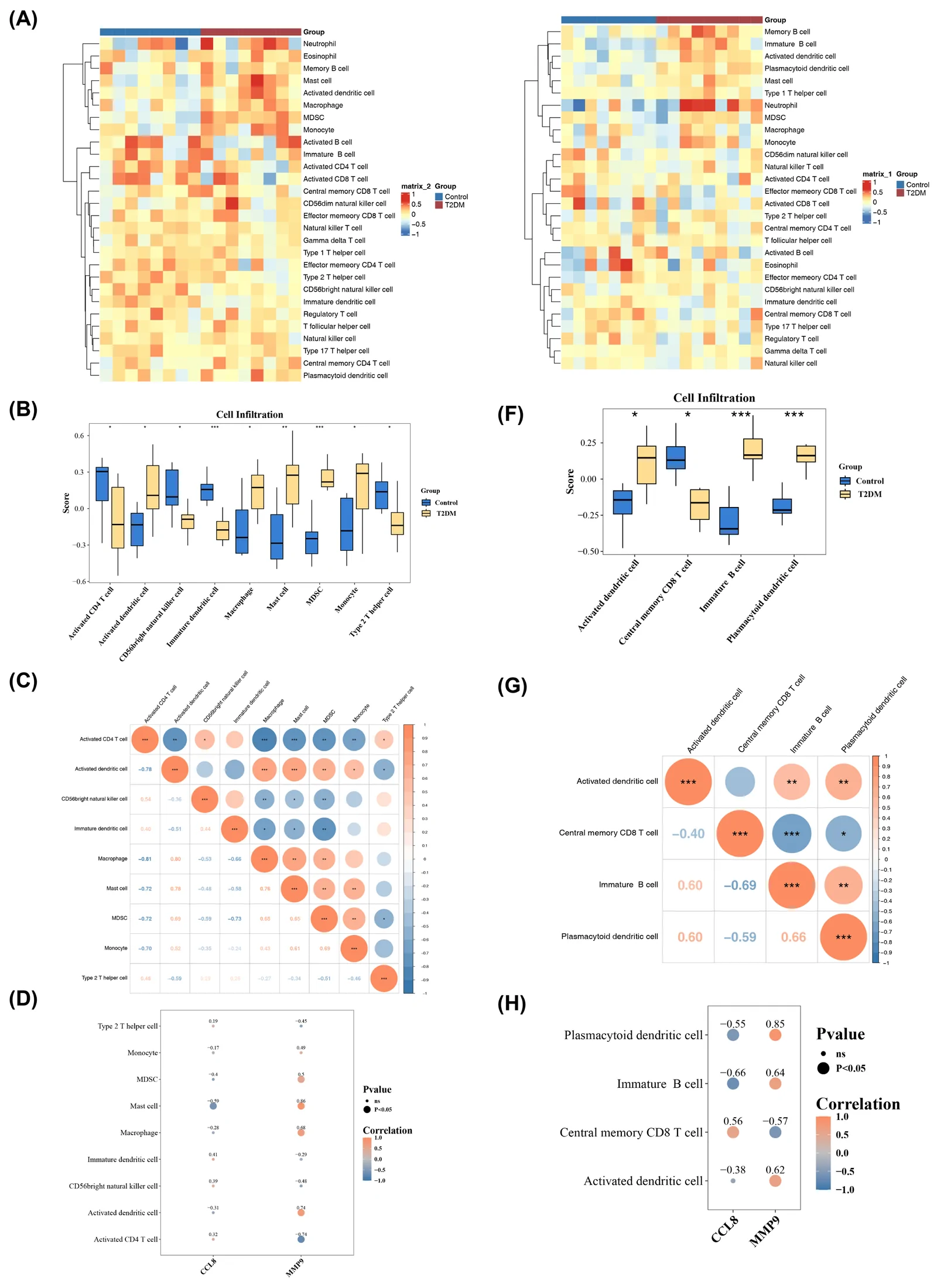
Immune cell infiltration in T2DM. (A) The heatmap shows the immune cell infiltration in different samples within the GSE15932 dataset. (B) The boxplot compares the differences in immune cell infiltration between the T2DM group and the control group. (C) The heatmap displays the correlation coefficients between differentially infiltrating immune cells. (D) The heatmap presents the correlation coefficients between differentially infiltrating immune cells and biomarkers. (E–H) Immune infiltration analysis of the validation set included the following components: intergroup immune cell infiltration profiles (E), identification of differentially infiltrated immune cells between groups (F), correlation analysis among differentially infiltrated immune cells (G), and correlation analysis between differentially infiltrated immune cells and biomarkers (H). A correlation coefficient with an absolute value > 0.3 was considered a strong correlation, where a positive value indicates a positive correlation and a negative value indicates a negative correlation. n s, not significant, *p < 0.05, **p < 0.01, ***p < 0.001, ****p < 0.0001.

Reflecting the immunomodulatory nature of the SASP, the circulating immune profiles of T2DM and control groups were characterized within GSE15932 and GSE21321 dataset. Specifically, in the GSE15932 dataset, it was noted that the cell scores for populations such as myeloid-derived suppressor cells (MDSCs) and mast cells were comparatively elevated, whereas immature B cells demonstrated a significantly greater abundance in the control group (Fig. 5A). Notably, nine differentially abundant immune cells were identified in the T2DM group, such as macrophages and monocytes were significantly upregulated in the T2DM group, while activated CD4+ T cells, CD56bright natural killer cells, and type 2 T helper cells were significantly downregulated (Fig. 5B). Among the analyzed circulating immune cell interactions, a robust positive correlation was identified between activated dendritic cells and mast cells (cor = 0.78, *P* < 0.05). Conversely, a significant inverse relationship was detected between macrophages and activated CD4+ T cells (cor = −0.81, *P* < 0.05) (Fig. 5C). Additionally, a notable positive correlation was observed between the expression levels of *MMP9* and those of mast cells, macrophages, MDSCs, as well as activated dendritic cells (cor > 0.3, *P* < 0.05). Conversely, a significant negative correlation was identified with activated CD4+ T cells (cor = −0.74, *P* < 0.05). Notably, *CCL8* expression exhibited a significant negative correlation solely with mast cells (Fig. 5D). Similarly, in the GSE21321 dataset, a total of 4 differentially abundant immune cell subsets were identified between T2DM and control samples, including activated dendritic cells, central memory CD8+ T cells, immature B cells, and plasmacytoid dendritic cells (Figs. 5E–5F). Among these, activated dendritic cells showed consistent results with the GSE253502 dataset, exhibiting increased infiltration in T2DM. Activated dendritic cells were strongly positively correlated with immature B cells and plasmacytoid dendritic cells (cor = 0.60, *P* < 0.01), while strongly negatively correlated with central memory CD8+ T cells (cor = −0.40) (Fig. 5G). Consistently, *MMP9* was strongly positively correlated with activated dendritic cells (cor = 0.62, *P* < 0.05), whereas *CCL8* showed the opposite trend (cor = −0.38) (Fig. 5H). Interestingly, although distinct differentially abundant immune cell subsets were identified in the GSE21321 and GSE253502 datasets, the correlation trends between the biomarkers and these cells remained consistent: when *MMP9* exhibited a positive correlation with a specific immune cell subset, *CCL8* showed an inverse correlation trend. These findings indicate that the peripheral immune profile of T2DM patients exhibits certain heterogeneity, while the correlations between the biomarkers and these infiltrating immune cells are maintained consistently.

### Validation of *MMP9* in GSE280401 single-cell dataset

**Figure 6:**
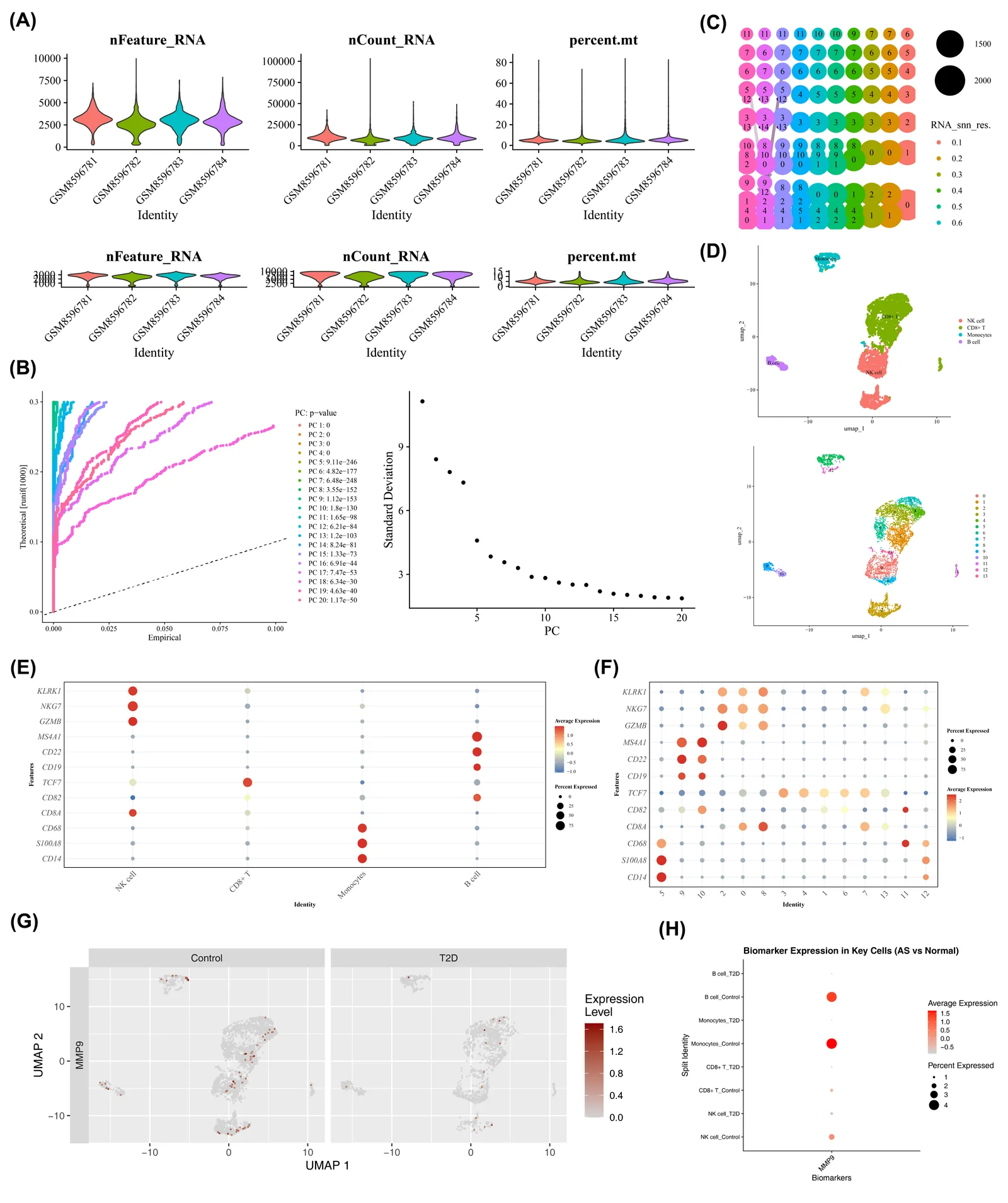
Single-cell RNA-seq profiling and biomarker validation in PBMCs from patients with T2DM and healthy controls ( GSE280401 dataset). (A) Quality control (QC) of single-cell RNA-seq data. Upper panel: Distribution of nFeature_RNA, nCount_RNA and mitochondrial gene percentage (percent.mt) before QC; Lower panel: Distribution of the above three indicators after strict QC filtration. (B) Identification of statistically significant principal components (PCs). Left panel: PCA JackStraw plot showing the *P*-value of each PC; Right panel: ElbowPlot displaying the standard deviation of PCs to determine the optimal PC number. (C) Optimization of cell clustering resolution, with the optimal clustering resolution determined as 0.5. (D) UMAP visualization of single-cell clustering results. Upper panel: Annotated four major immune cell subsets (NK cells, CD8+ T cells, B cells, Monocytes); Lower panel: Unannotated cell clustering distribution. (E) Dot plot of cell-type specific marker genes, presenting the average expression level and percentage of expressed cells for each marker gene in annotated cell subsets. (F) Dot plot of cell-type specific marker genes, showing the expression distribution characteristics of marker genes across distinct immune cell subsets. (G) UMAP visualization of MMP9 expression distribution in healthy control and T2DM groups. (H) Dot plot of MMP9 expression in key immune cell subsets, comparing the average expression level and percentage of expressed cells between T2DM and control groups.

A total of 7,792 original cells were acquired from the GSE280401 dataset, and 5,278 high-quality cells were retained after QC (Fig. 6A). PCA and ElbowPlot identified 14 statistically significant PCs (*P* < 0.05) (Fig. 6B). Cell clustering was optimized at a resolution of 0.5, and four major immune cell subsets were annotated, including NK cells, CD8+ T cells, B cells, and monocytes (Figs. 6C, 6D, Table S6). The marker genes (GZMB/NKG7/KLRK1 for NK cells, CD8A/CD82/TCF7 for CD8+ T cells, CD19/CD22/MS4A1 for B cells, CD14/S100A8/CD68 for monocytes) confirmed the reliability of cell annotation (Figs. 6E, 6F). *CCL8* was excluded during QC filtering, while *MMP9* showed stable expression in the single-cell dataset. UMAP visualization revealed that *MMP9* was predominantly distributed in monocytes, and its expression level was significantly higher in T2DM samples than in controls (Figs. 6G, 6H). This expression trend was highly consistent with the bulk RNA microarray data from GSE15932, GSE21321, and GSE253502, strongly supporting the stability and cell specificity of *MMP9* as a T2DM-related SASP biomarker.

To enhance the reliability of findings and address the requirement for robust external validation, the large-scale independent cohort GSE184050 (50 T2DM *vs.* 66 controls) was analyzed. Expression levels of *CCL8* and *MMP9* were compared between groups using the limma package. Results confirmed that the expression trends of *CCL8* and *MMP9* in GSE184050 were highly consistent with those in the training set (GSE15932) and previous validation sets (Fig. S1), supporting the stability of these candidate biomarkers across independent cohorts.

### Senescence signature scoring and correlation with *CCL8*/*MMP9*

**Figure 7:**
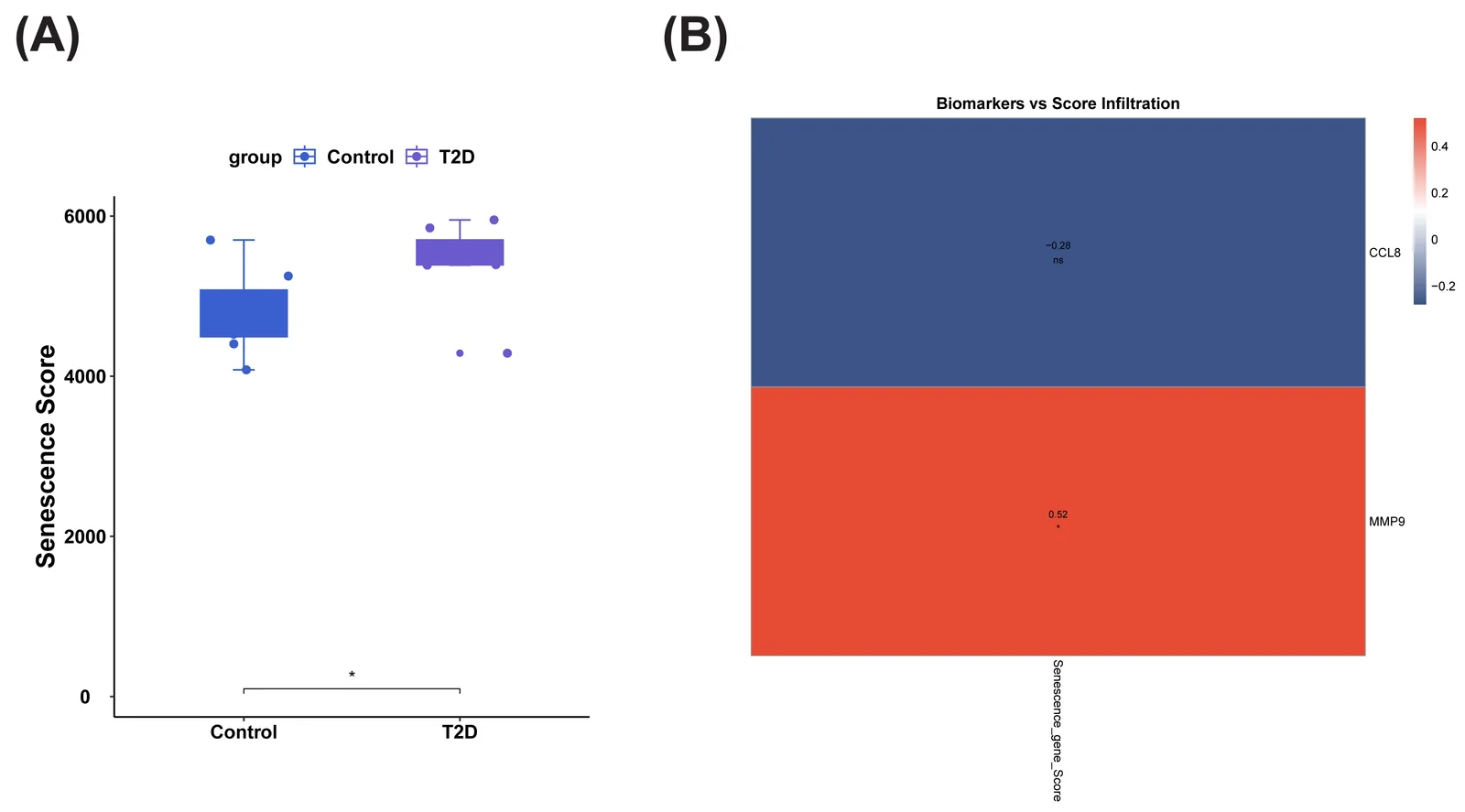
Senescence signature scoring and correlation with biomarkers. (A) Boxplot of senescence scores between T2DM and control groups. (B) Correlation heatmap between senescence scores and CCL8/MMP9 expression.

Following the standardized senescence scoring algorithm (Kohli et al., 2021), the senescence score of each sample was calculated. The Wilcoxon rank-sum test showed that the senescence score was slightly upregulated in the T2DM group compared with the control group (Fig. 7A). Correlation analysis revealed that *MMP9* expression was significantly positively correlated with the senescence score (cor = 0.52, *P* < 0.05), while no significant correlation was observed between *CCL8* and the senescence score (Fig. 7B). These results directly linked *MMP9* to cellular senescence progression in T2DM, rather than just inflammatory activation, and were consistent across all independent validation cohorts, confirming the rigor and reproducibility of our findings in line with top senescence biomarker studies.

### Association of *CCL8* and *MMP9* with senescent cell burden in T2DM

**Figure 8:**
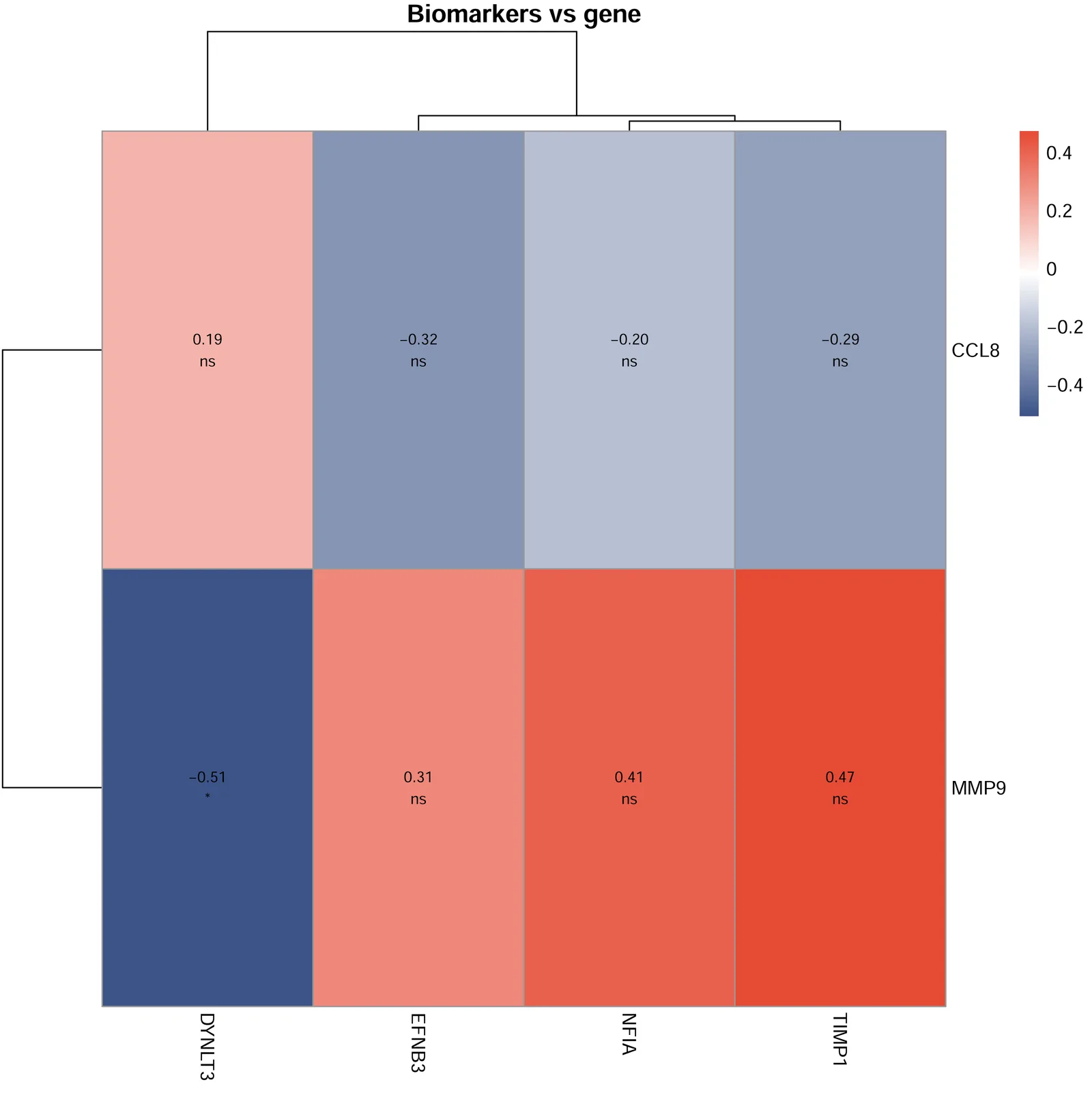
Correlation heatmap between biomarkers (CCL8/MMP9) and classic senescence marker genes. Red represents positive correlation, blue represents negative correlation; darker color indicates stronger correlation, and numbers represent correlation coefficients.

To verify the specific link between candidate biomarkers and senescent cells (rather than general inflammation), we quantified senescent cell burden using a 24-gene senescence signature. *MMP9* exhibited a strong positive correlation with the senescence marker gene DYNLT3 (cor = 0.47, *P* < 0.05), while no significant correlation was observed between *CCL8* and classic senescence marker genes (Fig. 8). These results indicated a significant correlation between MMP9 expression and senescent cell burden in T2DM, suggesting its potential as a senescence-associated candidate.

### Immune cell composition adjustment confirms *CCL8*/*MMP9* changes are independent of PBMC subset proportions

**Figure 9:**
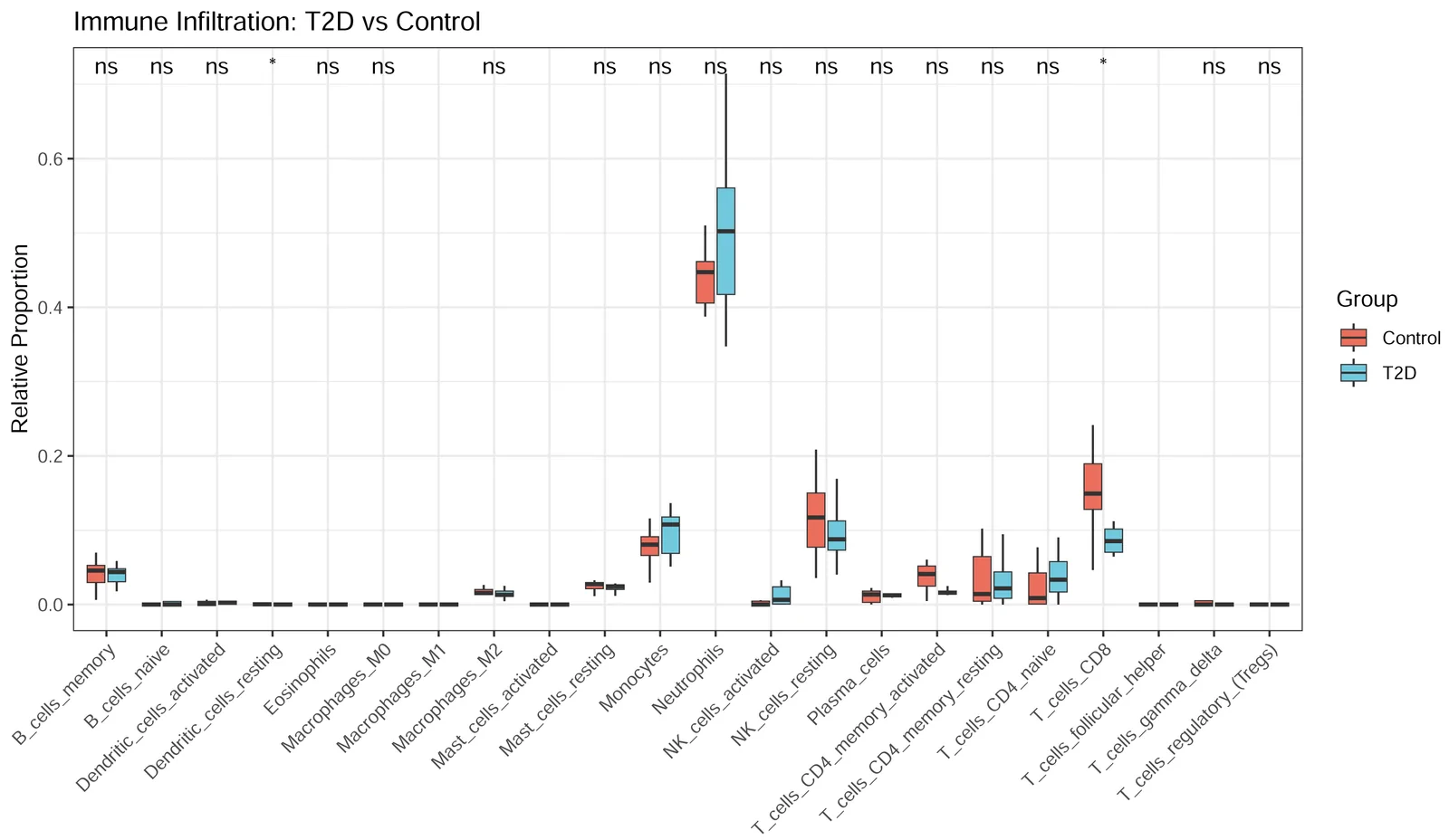
Comparison of immune cell subset proportions between T2DM and control groups in GSE15932 dataset estimated by CIBERSORT. Relative proportion of each immune cell subset is shown; ns, not significant (* *P* ≥ 0.05, Wilcoxon rank-sum test).

To address the technical concern of mixed PBMC population interference, CIBERSORT was used to calculate the proportions of 22 immune cell subsets in the GSE15932 dataset. Wilcoxon rank-sum test showed that most immune cell subsets exhibited no significant differences between T2DM and control groups (Fig. 9). ANCOVA adjustment with immune cell proportions as covariates confirmed that *MMP9* upregulation and *CCL8* downregulation in T2DM remained statistically significant (*P* < 0.05). Partial correlation analysis further verified that the correlations of *MMP9*/*CCL8* with senescence scores and T2DM status were still robust after controlling for immune cell proportions. These results confirmed that the expression changes of *CCL8* and *MMP9* were independent of PBMC subset frequency alterations, excluding false positive signals driven by immune cell proportion changes.

### Preliminary support for *CCL8* and *MMP9*

**Figure 10:**
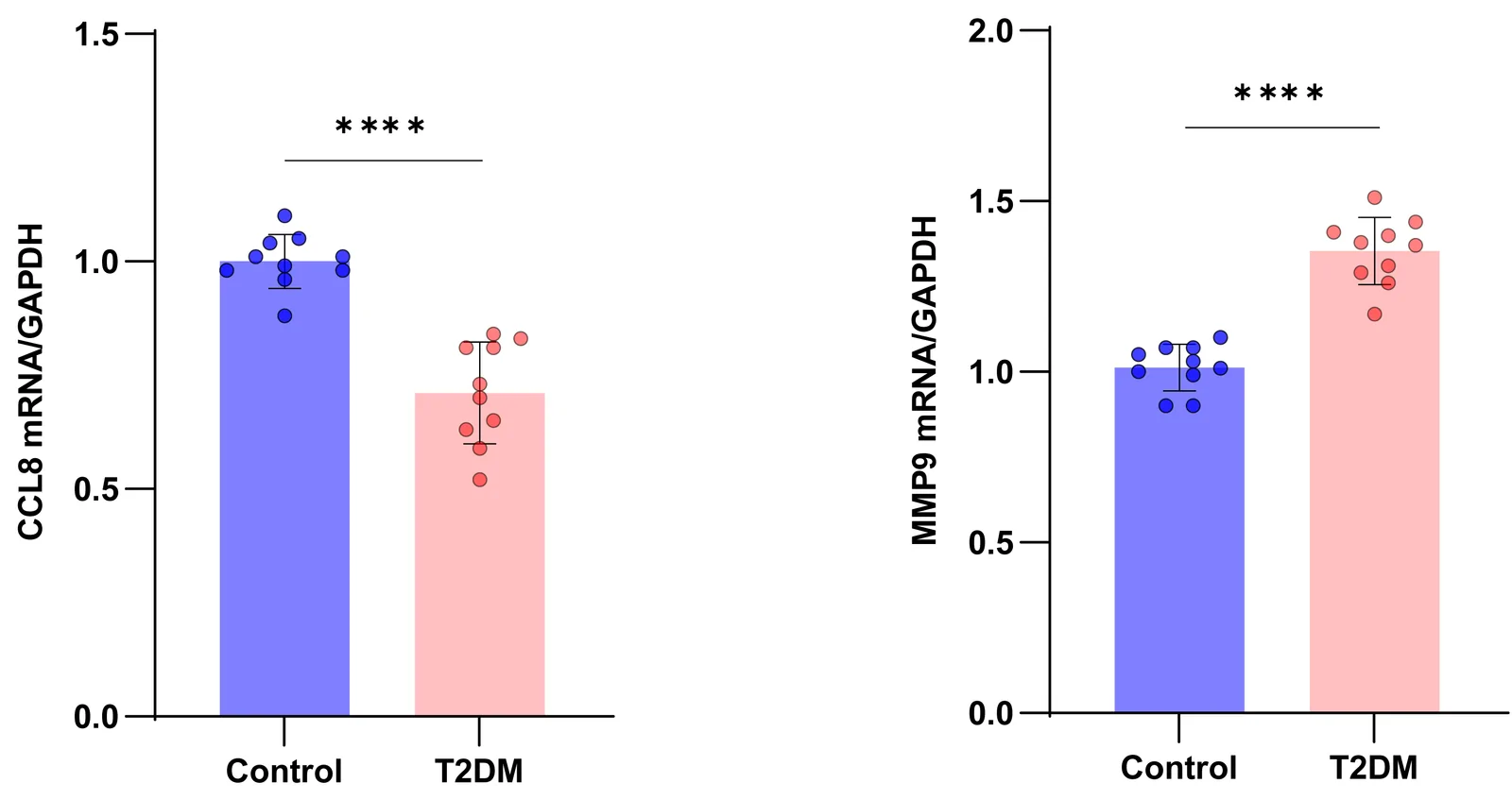
Expression of biomarkers in blood samples from clinical patients with T2DM. The expression levels of CCL8 and MMP9 in blood samples from the T2DM group and the control group were detected by RT-qPCR analysis. **** *P* < 0.0001.

The results obtained from RT-qPCR analysis provided preliminary exploratory support of *CCL8* and *MMP9* in the blood samples. Specifically, compared with the control group, *CCL8* mRNA levels were significantly decreased, whereas *MMP9* mRNA levels were significantly increased in blood samples from 10 individuals with T2DM (Fig. 10).

## Discussion

T2DM is a persistent metabolic disorder significantly associated with sustained low-grade inflammation (Sindhwani, Bora & Hazra, 2025). While the senescence-associated secretory phenotype (SASP, specifically secreted by senescent cells) is recognized as a driver of chronic inflammation (Sacco, Belloni & Latella, 2021; Zhou et al., 2020), the specific molecular signatures linking SASP to established T2DM remain to be fully elucidated. Notably, SASP-related genes extensively overlap with common immune inflammatory genes, and definitive evidence linking candidates to senescent cells is required to define *bona fide* SASP markers. In line with the paradigms of top senescence biomarker studies (Schafer et al., 2020; Basisty et al., 2020; Tanaka et al., 2020), our study adopted a rigorous “training set + three-layer independent validation set” strategy and introduced the standardized senescence signature scoring algorithm to improve analytical rigor. In this study, we employed machine learning techniques on transcriptomic datasets to identify senescent cell-linked SASP-related biomarkers. Our analysis identified *CCL8* and *MMP9* as key candidate genes that are significantly dysregulated in T2DM patients. Notably, both *CCL8* and *MMP9* have been previously reported in diabetes-related research, and the present study represents an integrative and confirmatory work rather than a mechanistically novel investigation. These genes were found to be enriched in pathways related to inflammation and immune response, specifically the “IL-17 signaling pathway” and ”TNF signaling pathway”, which aligns with previous findings on T2DM pathogenesis (Akash, Rehman & Liaqat, 2018; Elahi et al., 2024). Furthermore, immune infiltration analysis revealed a profound systemic immune imbalance in T2DM, characterized by the upregulation of myeloid cells and downregulation of lymphocytes, which correlated with the expression of these biomarkers.

The identification of *CCL8* and *MMP9* highlights the intersection of metabolic dysregulation and immune activation. Consistent with their roles as canonical SASP components secreted by senescent cells, these factors are known to mediate immune cell recruitment and extracellular matrix remodeling. In our study, *CCL8* and *MMP9* expression levels correlated significantly with the abundance of myeloid cells, including macrophages and monocytes. *MMP9* facilitates leukocyte migration *via* extracellular matrix degradation (Zhang et al., 2024), while *CCL8* acts as a potent chemoattractant for myeloid subsets (Zhang et al., 2020). Further correlation analysis demonstrated that *MMP9* was significantly and positively associated with the core senescence marker gene DYNLT3 (cor = 0.47, *P* < 0.05), confirming its specific linkage to cellular senescence rather than generic inflammation. Although these factors are established components of the SASP, their specific upregulation in T2DM blood samples suggests a broader role in the disease’s inflammatory milieu. For instance, *MMP9* elevation in T2DM has been linked to glycemic control and complications like nephropathy and delayed wound healing (Li et al., 2024; Zhang, Zhao & Zhu, 2020; Mohammadi et al., 2021), while *CCL8* is implicated in obesity-related inflammation and insulin resistance (Chen et al., 2019). Our findings support the notion that these molecules may serve as critical links between immune dysfunction and metabolic deterioration in T2DM. Notably, single-cell RNA-seq analysis of the GSE280401 dataset further validated that *MMP9* was specifically and significantly upregulated in circulating monocytes of T2DM patients, which was highly consistent with bulk transcriptomic results and clarified the cellular origin of *MMP9* overexpression at single-cell resolution. To further enhance the reliability of conclusions, the large-scale independent cohort GSE184050 (50 T2DM *vs.* 66 controls) was applied for external validation, and the expression trends of *CCL8* and *MMP9* were highly consistent across all datasets. Standardized senescence signature scoring (Kohli et al., 2021) further confirmed a significant positive correlation between *MMP9* expression and cellular senescence scores, directly tying this biomarker to SASP-associated senescence progression rather than general inflammatory activation alone. Additionally, CIBERSORT-based immune cell proportion deconvolution and covariate adjustment confirmed that the expression changes of *CCL8* and *MMP9* were independent of PBMC subset frequencies, excluding interference from altered immune cell proportions.

However, the interpretation of these findings requires a cautious approach regarding their specificity to cellular senescence. While we identified a robust correlation between *MMP9* and senescence markers/score, we acknowledge that this study does not provide direct *in situ* evidence of senescent cell origin (*e.g.*, SA-β-gal staining, p16/p21 immunostaining in tissues). Furthermore, several technical limitations must be acknowledged. First, the clinical blood sample size for RT-qPCR validation was relatively small and lacked the assessment of absolute protein expression levels, which only provides preliminary exploratory support and cannot substantiate diagnostic utility or biomarker robustness. Second, the public datasets used in this study lack complete clinical confounding factor information, including detailed medication usage, HbA1c levels and precise diabetes duration of T2DM patients, which may have potential impacts on the observed gene expression changes. Third, although initial analysis relied on bulk PBMC transcriptomic data, we have resolved the potential interference of immune cell subset proportions by performing CIBERSORT deconvolution, ANCOVA covariate adjustment, and partial correlation analysis, confirming that *CCL8*/*MMP9* dysregulation was independent of immune cell frequency changes. Fourth, to compensate for the platform limitations of the early microarray dataset GSE15932, we further introduced high-resolution single-cell RNA-seq dataset GSE280401 for validation, and the expression trends of *MMP9* were highly consistent across platforms, which effectively improved the reliability of the conclusions.Fifth, we have implemented the gold-standard senescence signature scoring algorithm and added correlation analysis with key senescence marker genes (*e.g.*, DYNLT3), which strengthened the mechanistic link between biomarkers and cellular senescence. Sixth, this study heavily relies on data quality and algorithm accuracy, which may lead to false-positive or false-negative results. Seventh, although we added the large-scale independent cohort GSE184050 for validation, further multi-center and larger-sample clinical studies are still required to enhance translational relevance. Furthermore, there is room for improvement in the rigor of analytical assumptions and the depth of the research.

Future research should address these limitations by expanding the sample size to multi-center, large cohorts covering different T2DM stages; performing ELISA assay for protein detection; conducting direct validation of senescent cell burden *via* SA-β-gal staining and p16/p21/LMNB1 expression detection; performing *in vivo* and *in vitro* experiments to confirm SASP activation from senescent cells; conducting flow cytometry to verify immune cell subset proportions and validate in silico deconvolution results; optimizing experimental design to eliminate the interference of mixed PBMCs; and improving analytical methods to distinguish SASP-specific from general inflammatory signatures. Future studies can also integrate senescence signature scoring and senescence gene correlation into clinical validation to quantify the correlation between biomarker levels and *in vivo* senescence burden. In addition, subsequent studies can combine spatial transcriptomics and *in vivo* tracing techniques to accurately locate the cellular sources of SASP factors in pancreatic tissues and peripheral blood, and further clarify the causal relationship between SASP activation and T2DM progression. This will help clarify the specific role of SASP in T2DM and validate the clinical value of CCL8 and MMP9 as diagnostic or therapeutic targets.

## Conclusions

Overall, these findings provide supportive evidence that CCL8 and MMP9 may represent SASP-related candidate biomarkers in T2DM and offer a systematic framework for screening and validating SASP-related candidates across independent cohorts. Further validation in larger cohorts and with protein- or cell-level assays is needed to determine biological mechanisms and clinical relevance.

##  Supplemental Information

10.7717/peerj.21729/supp-1 Supplemental Information 1 Expression analysis of key candidate genes in the large-scale independent validation cohort GSE184050 The x-axis shows gene names, and the y-axis shows gene expression levels. Red dots represent T2DM group, blue dots represent control group; ns, not significant.

10.7717/peerj.21729/supp-2 Supplemental Information 2 The complete list of 83 SASP-RGs collected from literature

10.7717/peerj.21729/supp-3 Supplemental Information 3 The complete list of DEGs identified in the GSE15932 dataset (T2DM vs. Control)

10.7717/peerj.21729/supp-4 Supplemental Information 4 GO enrichment analysis results for the 7 candidate SASP-RGs

10.7717/peerj.21729/supp-5 Supplemental Information 5 GSEA results for biomarkers * MMP9* and * CCL8*

10.7717/peerj.21729/supp-6 Supplemental Information 6 Predicted TFs targeting the biomarkers * MMP9* and * CCL8*

10.7717/peerj.21729/supp-7 Supplemental Information 7 Marker genes for cell type annotation in GSE280401

10.7717/peerj.21729/supp-8 Supplemental Information 8 MIQE checklist

10.7717/peerj.21729/supp-9 Supplemental Information 9 STROBE Checklist

10.7717/peerj.21729/supp-10 Supplemental Information 10 Scripts

10.7717/peerj.21729/supp-11 Supplemental Information 11 Raw results of PCR

## Abbreviations

The following abbreviations are used in this manuscript:

Abbreviation: **Full Term**
SASP: senescence-associated secretory phenotype
T2DM: type 2 diabetes mellitus
SASP-RGs: SASP-related genes
DEGs: differentially expressed genes
PBMC: peripheral blood mononuclear cell
FBG: fasting blood glucose
RT-qPCR: reverse transcription-quantitative polymerase chain reaction
GEO: gene expression omnibus
FC: fold change
GO: Gene Ontology
KEGG: Kyoto Encyclopedia of Genes and Genomes
PPI: protein-protein interaction
OOB: out-of-bag
RF: random forest
LASSO: least absolute shrinkage and selection operator
ROC: receiver operating characteristic
GSEA: Gene Set Enrichment Analysis
NES: normalized enrichment score
FDR: false discovery rate
MSigDB: molecular signatures database
miRNAs: microRNAs
TFs: transcription factors
BH: Benjamini-Hochberg
CCs: cellular components
MFs: molecular functions
BPs: biological processes
MDSCs: myeloid-derived suppressor cells
PAI-1: plasminogen activator inhibitor-1
DCs: dendritic cells

## Additional Information and Declarations

### Data Availability

The following information was supplied regarding data availability: The data is available in the Supplemental Files. The data is available at Zenodo: jia, . jiameng . (2026). Raw data of bioinformatics [Dataset]. Zenodo. https://doi.org/10.5281/zenodo.18240712
