# Supplementary material for: Integrative identification and preliminary validation of senescence-associated secretory phenotype-related candidate biomarkers in type 2 diabetes
Source: PeerJ. 2026 Sep 22;14:e21729. doi: 10.7717/peerj.21729 (PMC13614271; doi:10.7717/peerj.21729)
Supplement: Supplementary file 1 [file peerj-14-21729-s001.pdf]

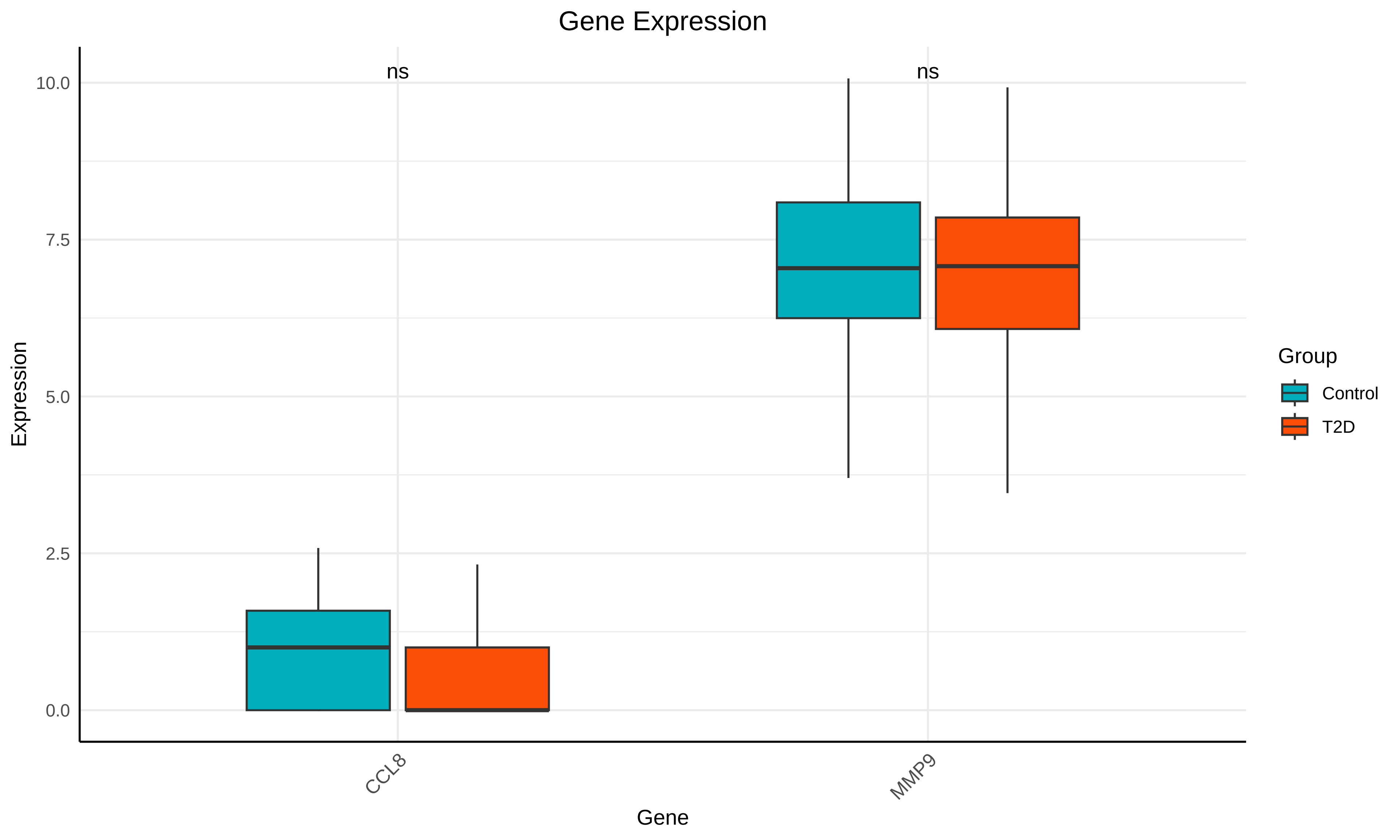
Gene Expression
ns
ns
10.0
7.5
5.0
2.5
0.0
Expression
CCL8
MMP9
Gene
Group
Control
T2D
